# Peculiar Clinical Manifestation in a Child With Self‐Limited Epilepsy With Autonomic Seizures (SeLEAS) Presenting in an All‐Four Position: A Case Report

**DOI:** 10.1155/crnm/6382812

**Published:** 2026-02-15

**Authors:** Niranjana Kesavamoorthy, Savitra Bandari

**Affiliations:** ^1^ JFK University Medical Center, 60 James Street, Edison, 08820, New Jersey, USA

**Keywords:** case report, focal autonomic seizures, Panayiotopoulos syndrome, SeLEAS

## Abstract

Self‐limited epilepsy with autonomic seizures (SeLEAS), previously known as Panayiotopoulos syndrome, presents with focal autonomic seizures in early childhood and is a self‐limited epilepsy syndrome. Previous articles have described the various manifestations of this syndrome, which are predominantly autonomic symptoms. In this article, we describe a child with an unusual initial presentation of SeLEAS. She was found resting on her elbows on all fours, a symptom that has not been previously reported and is different from the usually reported clinical presentations. This case underscores the importance of recognizing diverse clinical manifestations of SeLEAS to avoid misdiagnosis so that timely, appropriate management can be initiated.

## 1. Introduction

“Self‐limited epilepsy with autonomic seizures (SeLEAS), formerly known as Panayiotopoulos syndrome or early onset benign occipital epilepsy, is characterized by the onset in early childhood of focal autonomic seizures that are often prolonged.” SeLEAS occurs between 1 and 14 years of age, with most cases occurring between 3 and 6 years. Typical clinical presentations of SeLEAS include diverse autonomic features such as vomiting, dry heaving, sweating, pallor, pupillary dilation, and heart rate variability. To make a definitive diagnosis, autonomic focal seizures should be present [1]. Previous articles have reported different clinical presentations in children diagnosed with SeLEAS [2–5]. Although different clinical presentations have been reported, a child presenting in a prone position (presenting on all fours) is atypical, and it is important to recognize this manifestation to avoid misdiagnosis and initiate appropriate management. This presentation on all fours could be an instinctive response to retching or vomiting during sleep. Electroencephalogram (EEG) helps in the diagnosis of SeLEAS. Neuroimaging with MRI brain is considered in atypical presentations. In this article, we present a child with SeLEAS who exhibited an unusual clinical presentation that has not been reported before. We also describe the management approach taken in this case.

## 2. Case Description

**FIGURE 1:**
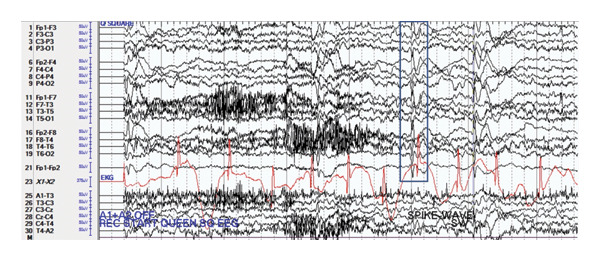
Interictal spike and wave discharges (indicated by the blue box).

A four‐year‐old, typically developing female child with no significant past medical history, presented with a new onset of seizure. She was taking a nap at school, and upon awakening, school staff reported that she was on all fours with her elbows down to the ground, with her neck in a hyperextended position. When trying to interact and call the patient, she was not responsive. School staff turned her over and noted the right upper extremity shaking that lasted approximately 1 minute. During this time, it was noted that her eyes were open, and the patient was unresponsive to others calling her name or other forms of stimulation. Subsequently, she had an episode of emesis. EMS was called, and she was transported to the ED. During her transport, the patient stared as if having a “glossed‐over look” on her face without much responsiveness or interaction with her parents. She was unresponsive for a long time and returned to her baseline after one and a half hours. Upon arrival at the ED, the patient gradually improved and became more interactive with her parents. She has never had any similar episodes in the past. The family denies noticing any prior history of staring without responsiveness. The family reports no significant illnesses or any head traumas recently, but she sustained head trauma in September 2022 when the patient fell 15 steps and struck her head. The CT head during that presentation did not reveal any intracranial abnormalities. The family reports she is reaching milestones within normal limits. No behavior and mood concerns were reported. No obstetric complications or pregnancy complications were noted. She was born at full‐term (40 weeks of gestational age). Family history was negative for seizures or neurological disorders. The neurological exam was nonfocal. She was subsequently admitted for video electroencephalogram (VEEG), which revealed bilateral parieto‐occipital and frontal spike and wave discharges, more prominent on the right when than that on the left, consistent with the diagnosis of SeLEAS (Figure 1). She was loaded with IV levetiracetam 20 mg/kg and started on maintenance with levetiracetam 10 mg/kg/dose twice daily (levetiracetam 200 mg twice daily), and she was discharged home. Given subtherapeutic levetiracetam levels during follow‐up appointment, the levetiracetam dose was increased from 200 mg twice daily to 300 mg twice daily. The MRI brain did not show any abnormalities.

## 3. Discussion

Panayiotopoulos first described vomiting as an ictal manifestation in children, which led to the discovery of Panayiotopoulos syndrome. In those children, EEG findings were present in the occipital region. It is a self‐limiting childhood epilepsy syndrome that is characterized by autonomic symptoms such as nausea, vomiting, pallor, cyanosis, hypersalivation, pupillary changes, cephalic auras, thermoregulatory, cardiovascular, gastrointestinal issues, incontinence of urine, and loss of consciousness [5–7]. It typically occurs in association with sleep [8], as seen in our patient also, where the child had seizures when waking up from her nap.

Previous articles have reported children with different clinical features such as laughter followed by unconsciousness, hiccups, eye deviation, cough followed by vomiting, hyperthermia, vertiginous features, seizures occurring during sleep, and cardiac arrest [3]. The article by Ferrie et al. described a patient with Panayiotopoulos syndrome with atypical features such as atonic seizures, atypical absences, and a reduction in intellectual performance [2]. There has been an association between SeLEAS patients with neurocognitive issues and anxiety. Neuropsychological symptoms have been reported to be more prominent following status epilepticus [9]. SeLEAS mimics several other neurological conditions such as migraine, syncope, and encephalitis [4]. In our case, the child was found on all fours with neck hyperextended, which is an unusual presentation and has not been previously reported. She had emesis during the seizure episode, as with the previously reported patients with SeLEAS, and had associated focal motor seizures.

Characteristics of the EEG findings include occipital spike and wave or high‐voltage sharps, but extra occipital spikes can also occur [8]. The VEEG of our patient showed bilateral parieto‐occipital spikes, consistent with the characteristic EEG findings. It is important to note that SeLEAS is the most common reason behind nonfebrile nonconvulsive status epilepticus in children [10]. Hence, it is important to recognize the clinical features to differentiate it from other conditions to provide the appropriate management.

SeLEAS is a self‐limiting disorder, and antiseizure medications are indicated when the child has frequent seizures, age of onset before 4 years of age, and quality of life is affected [11]. Levetiracetam has been reported as an effective antiseizure medication in patients with SeLEAS [12]. Other treatment options include carbamazepine, sodium valproate, and clobazam [13]. Levetiracetam has a favorable side effect profile and is hence used in our patient. Our case report gives a good clinical picture of the child’s presentation; however, a video of the event is not available, which is a limitation.

## Author Contributions

Niranjana Kesavamoorthy: writing–original draft, conceptualization, formal analysis, and investigation.

Savitra Bandari: writing–review and editing, supervision, and conceptualization.

All authors agree to be accountable for the content and conclusions of the article.

## Funding

The authors received no specific funding for this work.

## Disclosure

No third‐party services were involved in the research or manuscript preparation who are not listed as an author and have not been acknowledged.

## Consent

Signed consent for this case report was obtained from the patient’s legally authorized representative (LAR).

## Conflicts of Interest

The authors declare no conflicts of interest.

## Supporting Information

CARE Checklist of information to include when writing a case report

## Supporting information


**Supporting Information** Additional supporting information can be found online in the Supporting Information section.

## Data Availability

Data sharing is not applicable to this article as no datasets were generated or analyzed during the current study.
